# Fluorescence Methods Applied to the Description of Urea-Dependent YME1L Protease Unfolding

**DOI:** 10.3390/biom10040656

**Published:** 2020-04-23

**Authors:** Sydney Moore, Alyssa Pickens, Jessica L. Rodriguez, Justin D. Marsee, Justin M. Miller

**Affiliations:** 1 Department of Chemistry, Middle Tennessee State University, 1301 East Main Street, Murfreesboro, TN 37132, USA; smm2au@mtmail.mtsu.edu (S.M.); alp7p@mtmail.mtsu.edu (A.P.); jdm2am@mtmail.mtsu.edu (J.D.M.); 2 Vanderbilt University Medical Center, 1211 Medical Center Drive, Nashville, TN 37232, USA; jessica.l.rodriguez@vumc.org; 3 Middle Tennessee State University, Molecular Biosciences Program, 1301 East Main Street, Murfreesboro, TN 37132, USA

**Keywords:** YME1, mitochondria, ATP-dependent protease, AAA+ protease, protein unfolding, proteostasis

## Abstract

ATP-dependent proteases are ubiquitous across all kingdoms of life and are critical to the maintenance of intracellular protein quality control. The enzymatic function of these enzymes requires structural stability under conditions that may drive instability and/or loss of function in potential protein substrates. Thus, these molecular machines must demonstrate greater stability than their substrates in order to ensure continued function in essential quality control networks. We report here a role for ATP in the stabilization of the inner membrane YME1L protease. Qualitative fluorescence data derived from protein unfolding experiments with urea reveal non-standard protein unfolding behavior that is dependent on [ATP]. Using multiple fluorophore systems, stopped-flow fluorescence experiments demonstrate a depletion of the native YME1L ensemble by urea-dependent unfolding and formation of a non-native conformation. Additional stopped-flow fluorescence experiments based on nucleotide binding and unfoldase activities predict that unfolding yields significant loss of active YME1L hexamers from the starting ensemble. Taken together, these data clearly define the stress limits of an important mitochondrial protease.

## 1. Introduction

ATP-dependent proteases represent a ubiquitous class of molecular machines tasked with the maintenance of protein quality control under varied stress conditions [1,2,3,4,5]. These macromolecular complexes are responsible for the regulated removal of protein substrates recognized as misfolded, aggregated, or degradation-tagged [1,3,6,7]. Representative family members include FtsH, Lon, ClpXP, ClpAP, and the eukaryotic 26S proteasome. All ATP-dependent proteases share a common overall architecture wherein a ring-shaped AAA+ (ATPases Associated with various cellular Activities) [8,9,10] motor component drives ATP-dependent unfolding and translocation of specific protein substrates into a bulk solvent-inaccessible proteolytic compartment for degradation [3,4,7,11,12,13,14,15]. AAA+ protease-catalyzed protein substrate unfolding and translocation requires AAA+ domain motions that are coupled to nucleotide hydrolysis [13,16,17,18]. For this reason, conformational dynamics for these protease machines represent a significant driving force in dictating stress response and functional output.

The successful execution of protein quality control function by an ATP-dependent protease requires structural stability under stress conditions that may drive instability and/or loss of function for protein substrates. Mitochondria represent a unique environment where adaptation to stress is particularly important. For example, chronic kidney disease may challenge mitochondria by the dual-presentation of uremic and oxidative conditions [19,20,21]. Chronic kidney disease is associated with increased oxidative stress and the production of pathogenic levels of uremic toxins that include urea, cyanate, p-cresol sulfate, indoxyl sulfate, etc. [22,23,24,25]. Therefore, protein quality control components in mitochondria must be adapted to retain stability under conditions that may include multiple, different types of simultaneous stressors. Failure to do so may result in the decline of overall mitochondrial function due to associated loss of function for related quality control systems.

The first layer of mitochondrial quality control is controlled by ATP-dependent proteases that catalyze the selective removal of protein substrates recognized as being degron-tagged, misfolded, or aggregated [3,11,26,27]. Protein quality control at the mitochondrial inner membrane involves contributions from FtsH-like zinc-metalloproteases embedded in the membrane and includes AFG3L2, YME1L, paraplegin, etc. [28]. Based on our previous work, the conformational behavior exhibited by a recombinant YME1L construct [29] depends on the concentration of available nucleotide [30]. Oxidative conditions are known to promote depletion of the nucleotide pool due to a decrease in mitochondrial respiratory function. Moreover, uremic conditions are known to also promote oxidative stress. From this, we asked: how does YME1L respond to changing nucleotide availability when exposed to chemical stress? We report here a role for ATP in the stabilization of recombinant cc-hex YME1L [29] against urea denaturation. Using a combination of fluorescence-based equilibrium and kinetic methods, we report here that a subset of the native YME1L ensemble undergoes unfolding in response to acute urea exposure. The data presented here indicate that the most of the starting YME1L ensemble becomes non-functional in response to urea treatment. However, the presence of ATP dampens this response by protecting against initial unfolding. Urea-treated YME1L binds nucleotide with decreased apparent rate constants relative to untreated samples. Apparent time course amplitudes suggest decreased concentrations of active enzyme under conditions including urea relative to non-stress conditions. Equilibrium titration experiments were performed to observe the impact of unfolding from a thermodynamic perspective. Experiments involving incubation of YME1L in the presence of varied nucleotide concentrations ranging from 0 to 2500 µM support a model wherein the native state is stabilized against urea denaturation when nucleotide is bound. Together, the data reported here shed light on the contribution of nucleotide to YME1L stability under conditions of urea stress.

## 2. Materials and Methods

### 2.1. Materials

All solutions were prepared with reagent-grade chemicals in double-distilled water produced from a Purelab Ultra Genetic System (Siemens Water Technology). Adenosine triphosphate (ATP) and 8-anilinonaphthalene-1-sulfonic acid (ANS) were obtained commercially from Sigma-Aldrich (St. Louis, MO, USA). MANT-ATP (2’/3’-O-(N-methylanthraniloyl) adenosine 5’-triphosphate) was prepared as described previously and purified using reverse-phase chromatography [31]. Product MANT-ATP was characterized by thin-layer chromatography and by mass spectrometry. MANT-ATP and ATP concentrations were determined spectrophotometrically using extinction coefficients ε_255_ = 2.33 × 10^4^ M^−1^ cm^−1^ and ε_260_ =1.54 × 10^4^ M^−1^ cm^−1^, respectively. 

All genes were synthesized and each cloned into the pET-24a(+) vector commercially by Genscript (Piscataway, NJ, USA). Soluble YME1L was expressed as the previously described cc-hex YME1L construct with the addition of an N-terminal His_6_-SUMO tag [29,30]. YME1L was purified as previously described [30] and protein concentration was determined prior to use in buffer H150 (25 mM HEPES (pH = 7.5 at 25 °C), 150 mM NaCl, 10% glycerol, 2 mM 2-mercaptoethanol, and 0.5 mM ZnCl_2_) using extinction coefficients ε_280_ = 3.29 × 10^4^ M^−1^ cm^−1^. Briefly, cc-hex YME1L was overexpressed in BL21(DE3)-competent *Escherichia coli* cells with an N-terminal His_6_-SUMO tag cleavable by treatment with the Ulp1 protease. After cell lysis and clarification, soluble material was incubated with Ni-nitriloacetic acid (Ni-NTA) in batch format to allow for His_6_-tag binding to solid-phase resin. His_6_-SUMO-YME1L eluted from Ni-NTA resin was then incubated overnight at 4 °C with His_6_-Ulp1 protease for tag removal. Cleaved His_6_-SUMO tag and His_6_-Ulp1 were removed by additional incubation with Ni-NTA resin. In some instances, we have utilized additional ion-exchange purification methods to remove residual Ulp1 and His_6_-SUMO tag. All ion-exchange purification steps were performed using a HiPrep Q FF 16/10 anion-exchange column (GE Healthcare, Chicago, IL) previously equilibrated in 25 mM Tris pH = 8.3, 10 mM NaCl, 20% glycerol, and 10 mM 2-mercaptoethanol. Cleaved cc-hex YME1L was eluted from the anion-exchange material over 8 column volumes using a linear gradient varying [NaCl] from 0 to 1 M. All protein was determined to be greater than 95% pure by coomassie staining techniques.

### 2.2. Methods

#### 2.2.1. Stopped-Flow Fluorescence Assays

**Figure 1:**
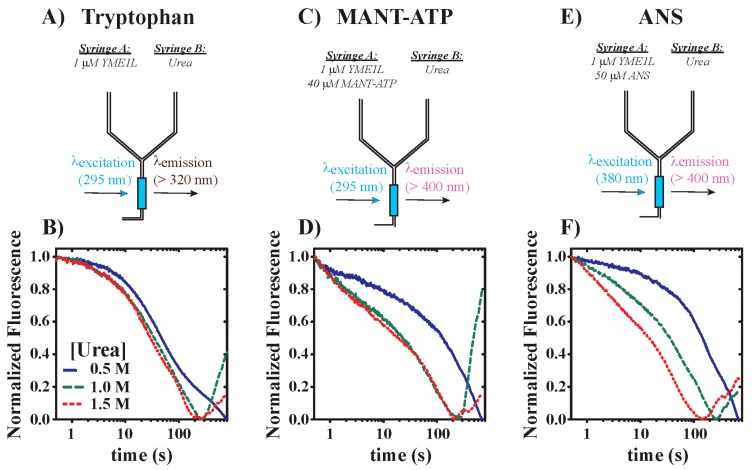
(**A**) Schematic representation of stopped-flow unfolding experiments reporting on tryptophan emissions. Syringes A and B contain 1 µM YME1L and varied urea concentrations (indicated in text), respectively. The contents of the two syringes are rapidly mixed in the stopped-flow spectrophotometer and YME1L is excited at λ_EX_ = 295 nm. Emissions are observed above 320 nm with a 320 nm-long pass filter. (**B**) Fluorescence time courses reporting on tryptophan emissions for urea-dependent YME1L unfolding. All urea concentrations represent final mixing conditions. (**C**) Schematic representation of preincubation stopped-flow unfolding experiments reporting on MANT-ATP emissions. Syringe A contains 1 µM YME1L and 40 µM MANT-ATP. Syringe B contains varied urea concentrations indicated in text. (**D**) Fluorescence time courses reporting on MANT-ATP emissions for urea-dependent YME1L unfolding. The contents of the two syringes are rapidly mixed in the stopped-flow spectrophotometer and YME1L is excited at λ_EX_ = 295 nm. Emissions are observed above 400 nm with a 400 nm-long pass filter. (**E**) Schematic representation of preincubation stopped-flow unfolding experiments reporting on ANS emissions. Syringe A contains 1 µM YME1L and 50 µM ANS. Syringe B contains varied urea concentrations indicated in text. (**F**) Fluorescence time courses reporting on ANS emissions for urea-dependent YME1L unfolding. The contents of the two syringes are rapidly mixed in the stopped-flow spectrophotometer and YME1L is excited at λ_EX_ = 380 nm. Emissions are observed above 400 nm with a 400 nm-long pass filter.

All stopped-flow fluorescence experiments were performed using an Applied Photophysics SX.20 stopped-flow fluorometer (Letherhead, UK). All reactions were performed at 25 °C in buffer H150 (25 mM HEPES, pH = 7.5 at 25 °C, 150 mM NaCl, 10% glycerol, 2 mM 2-mercaptoethanol, and 0.5 mM ZnCl_2_). Protein unfolding experiments were performed by rapid mixing of the contents of Syringes A and B, as schematized in Figure 1. For protein unfolding experiments, Syringes A and B contained 1 µM YME1L and defined concentrations of urea indicated in text, respectively. Modified experimental designs required preincubation of 1 µM YME1L with either 40 µM MANT-ATP or 50 µM ANS in Syringe A for 25 min at 25 °C to establish binding equilibrium. Prior to mixing, both solutions were incubated for an additional 10 min at 25 °C in the stopped-flow instrument to establish thermal equilibrium. Additional incubation of either solution had no effect on the observed fluorescence time courses. YME1L tryptophan residues were excited at λ_EX_ = 295 nm and fluorescence emissions were observed above 320 nm using a 320 nm-long pass filter. An excitation wavelength of λ_EX_ = 295 nm has been utilized here to allow for specific excitation of tryptophan residues without concern for contaminating the signal from tyrosine residues that would otherwise become excited at shorter excitation wavelengths. Nucleotide dissociation experiments utilized energy transfer between tryptophan residues excited at λ_EX_ = 295 nm and MANT-ATP by using a 400 nm-long pass filter. ANS dissociation experiments involved direct fluorophore excitation at λ_EX_ = 380 nm and emissions above 400 nm by using a 400 nm-long pass filter. All kinetic traces shown represent the average of at least four individual determinations. 

Nucleotide binding experiments were performed based on our previously reported protocol [30]. As shown schematically in Figure 5A, Syringes A and B contained 1 µM YME1L and 40 µM MANT-ATP, respectively, but in the presence of [urea] spanning from 0 to 4 M. All experiments were performed in buffer H150 lacking MgCl_2_ under pseudo-first-order conditions with [MANT-ATP] in 40-fold excess of [YME1L]. Final concentrations of YME1L and urea are described in text. As described above, YME1L tryptophan residues were excited at λ_EX_ = 295 nm and fluorescence emissions were observed above 400 nm with a 400 nm-long pass filter. All kinetic traces shown represent the average of at least five individual determinations. Averaged time courses were subjected to non-linear least squares (NLLS) analysis using a double exponential function.

#### 2.2.2. Equilibrium Unfolding Experiments

Equilibrium unfolding experiments were carried out in H150 buffer supplemented with 100 mM EDTA (ethylenediaminetetraacetic acid). Incubation was performed at 25 °C by incubating independent solutions overnight containing 0.5 µM YME1L in the presence of 0, 20, 500, or 2500 µM ATP and urea concentrations ranging from 0 to 6 M. Stock urea solutions were prepared in buffer H150 at pH = 7.5, at 25 °C. All fluorescence measurements were collected on a Hitachi F-4500 fluorescence spectrophotometer. Samples were excited at 295 nm and emissions spectra collected by scanning from 310 to 450 nm. All tryptophan emissions spectra are normalized relative to conditions lacking supplemented urea. 

Additional experiments were performed in the presence of ANS. YME1L was incubated overnight at 25 °C and under identical conditions to those described above. After overnight incubation, ANS was introduced at a concentration equal to 50 µM for 5–10 min prior to data collection. Samples were excited at 380 nm and emissions spectra were collected by scanning from 400 to 500 nm. All ANS emissions spectra were normalized relative to conditions lacking supplemented urea. 

## 3. Results

### 3.1. Acute Urea Stress Drives YME1L Denaturation

The continued functionality of YME1L under stress conditions requires it to be stable upon acute exposure to various environmental stressors. Thus, we performed stopped-flow fluorescence experiments wherein 1 µM YME1L was rapidly mixed with varied concentrations of urea. Figure 1A illustrates this experimental design wherein the signal is derived from time-dependent changes in tryptophan fluorescence. Figure 1B shows representative time courses where conditions that include final mixing urea concentrations equal to 0.5, 1.0, or 1.5 M promote a decrease in tryptophan fluorescence. For [urea] = 1.0 and 1.5 M, a recovery in fluorescence signal is observed after ~200 s. However, [urea] = 0.5 M, the lowest concentration of urea, does not demonstrate signal recovery on this timescale, which is likely a consequence of a decreased apparent rate constant for the observed kinetic event. Moreover, we observe time-dependent tryptophan quenching, followed by signal recovery to a non-wild-type state in steady-state fluorescence experiments performed over a 2 h period (Figure S1) in the presence of 2 M urea. Figure S1B reveals YME1L species that exhibit tryptophan emissions at later time points greater than observed at the start of reaction, which predicts a unique tryptophan environment for the urea-treated versus native species. However, it is unclear whether this unique tryptophan solvent environment is the consequence of refolding to a functional state, formation of an unfolding intermediate species, aggregation to a non-functional state(s), or a mixture of them all. Complementary stopped-flow fluorescence experiments reporting on YME1L unfolding based on tryptophan emissions indicate that preincubation of YME1L in the presence of saturating [ATP] yields decreased time course amplitudes relative to apo conditions, consistent with nucleotide-dependent YME1L stabilization against unfolding (Figure S2). Figure S2B also highlights the observation of signal recovery for experiments performed in the presence of intermediate urea concentrations. Such behavior may suggest an unfolding model wherein the unfolded YME1L state, once formed, is more stable than alternative folded conformations at both low and high urea concentrations.

To determine whether YME1L unfolding observed in Figure 1B is accompanied by a loss of nucleotide binding activity and, therefore, represents a denatured state, we performed additional stopped-flow fluorescence experiments using a modified experimental design. As shown in Figure 1C, 1 µM YME1L was incubated in the presence of 40 µM MANT-ATP to promote formation of a nucleotide-bound hexamer. As schematized, the direct excitation of tryptophan residues at λ_EX_ = 295 nm allows for energy transfer to MANT-ATP when the two fluorophores are in close proximity. Homology modeling of the YME1L hexamer predicts that bound ATP is positioned adjacent to a tryptophan residue located in the lid subdomain of the AAA+ fold. Once the binding reaction reached equilibrium, the contents of syringes A and B were rapidly mixed to achieve final urea concentrations equal to 0.5, 1.0, or 1.5 M. Our rationale here is that urea-dependent YME1L unfolding would promote nucleotide dissociation, thereby leading to decreased MANT-ATP fluorescence. Consistent with this hypothesis, Figure 1D demonstrates that rapid mixing with urea promotes MANT-ATP dissociation with the primary evidence for this being a loss of MANT-ATP fluorescence. However, MANT-ATP is observed at later time points to exhibit a recovery in fluorescence, which may be the result of MANT-ATP association with previously unbound YME1L remaining in the native state, folding intermediates, or aggregated YME1L. Additional evidence for this phenomenon is derived from similar experiments performed by preincubation of 1 µM YME1L with 50 µM ANS prior to mixing with urea (Figure 1E). ANS is a commonly used fluorescent probe that binds to solvent-accessible patches of non-polar residues on the protein surface [32,33]. Such clusters of non-polar residues are commonly expected to become solvent inaccessible for the native state, but experience increased solvent accessibility for protein folding intermediates such as the molten globule. As such ANS is commonly utilized in the study of protein conformational changes associated with protein unfolding or ligand binding [34,35]. Figure 1F highlights the same trend wherein ANS fluorescence is quenched after ~200 s, but recovers at later time points. Taken together, these data clearly demonstrate that acute urea stress promotes YME1L denaturation associated with both a loss of structure sufficient to support either nucleotide or ANS binding. Moreover, we note that time courses reporting on tryptophan, MANT-ATP, and ANS fluorescence do not display identical kinetic behavior, which may result from the formation of intermediate species that differentially interact with each probe. Support for this conclusion is derived from our preliminary efforts to mathematically model the kinetic mechanism of YME1L unfolding, where at least 6–7 kinetic steps are required to describe each time course. For this reason, additional work will be needed to characterize the state(s) that populate after treatment with intermediate urea concentrations. 

### 3.2. YME1L Incubation with Urea Drives Alterations in Tryptophan Solvent Environments

**Figure 2:**
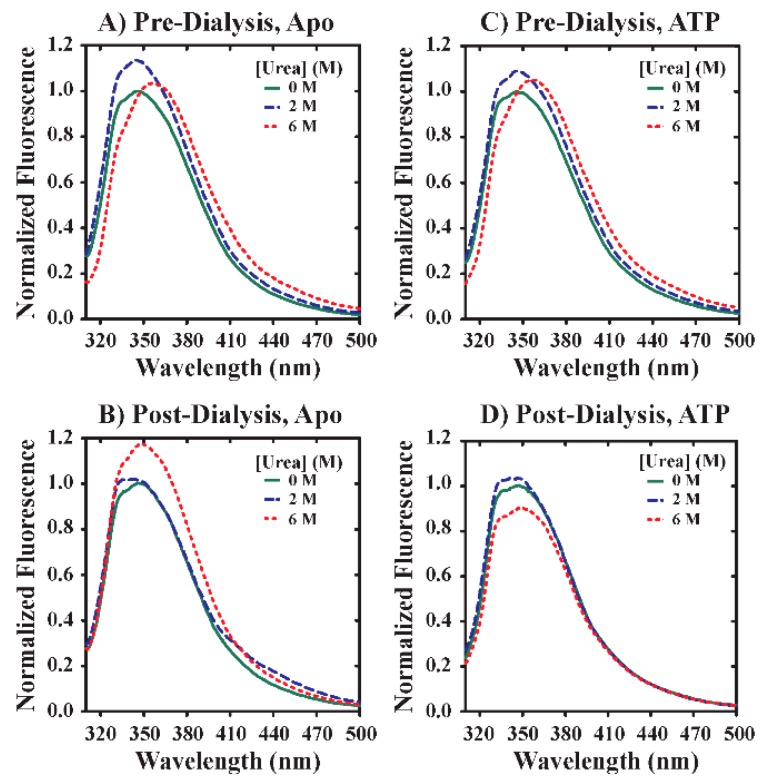
YME1L tryptophan emissions depend on [urea]. YME1L emissions spectra were collected in the presence of systematically varied concentrations of urea equal to 0 (solid green lines), 2 (blue dashed lines), and 6 M (red dashed lines) urea. Samples were prepared by overnight incubation at 25 °C of 0.5 µM YME1L with the indicated urea concentration in the absence (**A**,**B**) or presence (**C**,**D**) of 2500 µM ATP. Spectra were collected before and after dialysis to remove excess urea. Samples were excited at 295 nm and emissions spectra collected by scanning from 310 to 450 nm. All tryptophan emissions spectra are normalized relative to conditions lacking supplemented urea.

The kinetic experiments presented above indicate that the exposure of YME1L to urea drives unfolding that is followed by transition to a state(s) harboring a unique tryptophan solvent environment relative to the native species. This observation presented a fortuitous opportunity to utilize YME1L tryptophan emission properties to observe the impact of urea on protein folding under equilibrium conditions. For this reason, a series of equilibrium unfolding experiments were performed that reported on changes in tryptophan fluorescence in the presence of varied [ATP] and [urea]. YME1L (0.5 µM YME1L) was incubated in urea overnight in the presence of 0 or 2500 µM ATP and 100 mM EDTA. EDTA has been included to prevent metal-dependent proteolysis that may otherwise occur during extended incubations. Figure 2A highlights the observation of asymmetric emissions spectra in the absence of urea displaying emissions maxima at 330 and 350 nm, as confirmed by second derivative test (Figure 2A, solid green line). Comparison of incubation conditions reveals an ~ 15% increase in total fluorescence intensity when urea concentration is increased from 0 (Figure 2A, solid green line) to 2 M (Figure 2A, blue dashed line). An additional increase in [urea] to 6 M yields a shift in peak maximum to 360 nm (Figure 2A, red dotted line). Qualitative inspection of tryptophan emissions spectra collected in the presence of 2500 µM ATP confirm that this general trend occurs independent of [ATP] (Figure 2C). Taken together, these data suggest that urea drives a change in YME1L structure that alters the local solvent environment for tryptophan residues. 

Thermodynamic treatment of equilibrium unfolding data as described above requires knowledge of the reversibility of YME1L unfolding. To examine this question, we performed a series of experiments wherein YME1L tryptophan emissions spectra were collected in the presence of 0, 2, and 6 M urea and then again after denaturant removal by dialysis. Figure 2A,B present tryptophan emissions spectra collected before and after dialysis of 0.5 µM apo YME1L into fresh H150 buffer (25 mM HEPES, pH = 7.5 at 25 °C, 150 mM NaCl, 10% glycerol, 2 mM 2-mercaptoethanol, and 0.5 mM ZnCl_2_) supplemented with 100 mM EDTA. Prior to dialysis, all observed spectra are shown as Figure 2A. After extensive dialysis of urea-treated samples, Figure 2B demonstrates that YME1L unfolding is not reversible based on the observation of incomplete superimposition of emissions spectra collected for samples treated with 2 and 6 M urea. For YME1L samples previously treated with 2 M urea, denaturant removal allows for refolding to a state that yields a tryptophan solvent environment that is similar, but non-identical, to non-treated YME1L (Figure 2B, blue dashed line). A similar observation is noted when this procedure is repeated in the presence of 2500 µM ATP (Figure 2D, blue dashed line). Treatment of YME1L with 6 M urea appears to promote the irreversible formation of a YME1L conformational ensemble that is significantly different with respect to tryptophan solvent environment relative to post-dialyses samples treated with lower [urea]. However, all post-dialysis samples display emissions maxima at ~350 nm independent of urea concentration, thereby providing evidence of similar solvent accessibility for tryptophan residues in each case [36]. The observation of incomplete reversibility for YME1L unfolding across the full range of urea concentrations examined limits the application of common thermodynamic analysis methods such as the Linear Extrapolation Method [37,38]. 

**Figure 3:**
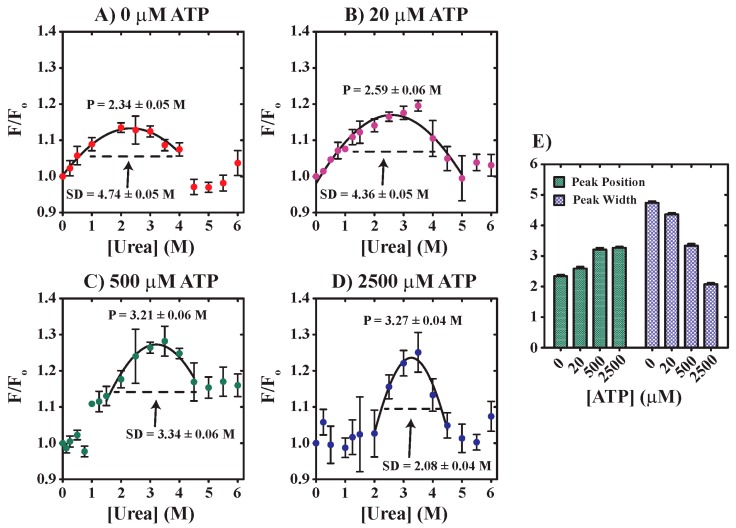
Dependence of normalized fluorescence observed at 350 nm on [urea] in the absence (**A**, red spheres) or presence of 20 μM (**B**, purple spheres), 500 μM (**C**, green spheres), and 2500 μM (**D**, blue spheres) ATP. The continuous line is the result of a non-linear least squares (NLLS) fit to a Gaussian function, with the maximum peak position, P, and standard deviation, SD, estimated as indicated. All data represent average values determined from at least three independent experiments. Error bars indicate ± standard deviation. (**E**) Summarized NLLS parameters presented in Figure 3A–D.

Given the observation of [urea]-dependent differences in the emissions behavior of YME1L tryptophan residues, we performed additional equilibrium unfolding experiments with the goal of understanding of the impact of nucleotide in YME1L stabilization. YME1L (0.5 µM YME1L) was incubated overnight at 25 °C in the presence of 0–6 M urea and varied [ATP]. The resultant data are plotted as normalized fluorescence derived from observed emissions recorded at 350 nm versus [urea] in Figure 3A–D. These data allow for the spectroscopic identification of three distinct tryptophan populations observed across a range of urea concentration from 0 to 6 M that must differ in local solvent environment. We refer here to these three populations as the native (N), intermediate (I), and terminal (T) states. We note here that each population is likely not homogenous and that these data represent only the average fluorescence behavior of the population. Rationale for this classification is based on [urea]-dependent changes in tryptophan emissions relative to conditions lacking urea, F/F_o_. Reference to Figure 3A demonstrates that F/F_o_ from 0 to 4 M urea for apo YME1L can be described by a Gaussian function with maximum position at 2.34 ± 0.05 M urea, which steeply decreases to an average of F/F_o_ = 0.99 ± 0.03 for [urea] > 4 M. Incubation in the presence of 20 µM ATP yields the same behavior, though with maximum F/F_o_ observed at 2.59 ± 0.06 M urea (Figure 3B). We have previously reported dissociation equilibrium constants for YME1L binding to nucleotide as 20 ± 7 µM and 74 ± 33 µM [30]. From this, we reason that the conditions presented in Figure 3B likely represent a mixture of bound and free YME1L ligation states. In contrast, Figure 3C,D reveal additional stability under conditions favoring saturated nucleotide binding such that the native state is stable in the presence of ~1 or 2 M urea when incubated with 500 or 2500 µM ATP, respectively. For these conditions, the intermediate population is described by a Gaussian function with a maximum peak position at 3.21 ± 0.06 M and 3.27 ± 0.04 M urea in the presence of 500 and 2500 µM ATP, respectively. Moreover, the peak width observed in Figure 3A–D is observed to decrease with increasing ATP concentration. However, the terminal state is fully populated in the presence of 5 M urea regardless of ATP concentration. All NLLS parameters are summarized in Figure 3E and demonstrate that both the maximum peak position and the peak width exhibit [ATP]-dependent behavior. These observations are of physiological relevance, since urea concentrations in renal inner medulla reach 500–600 mM [39,40].

**Figure 4:**
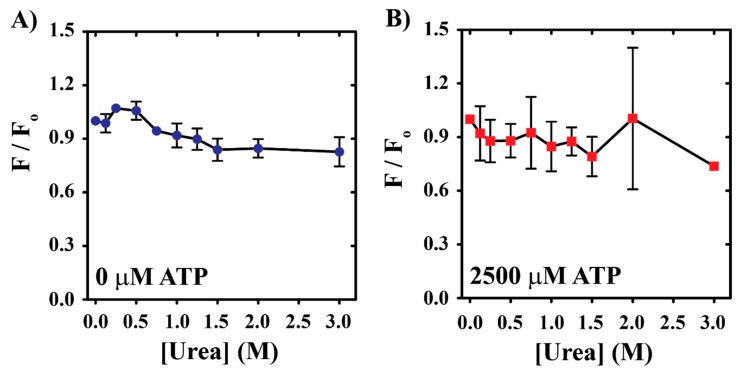
Dependence of normalized ANS fluorescence observed at 480 nm on [urea] in the absence (**A**, blue spheres) or presence of 2500 µM (**B**, red squares) ATP. All data represent average values determined from at least three independent experiments. Error bars indicate ± standard deviation.

The observation of constant tryptophan emissions that fluctuate about a baseline value for conditions including [ATP] > 500 µM and [urea] < 1–2 M suggests nucleotide-dependent YME1L stabilization. However, these data only highlight the similarity in tryptophan solvent environments promoted by each condition. Based on this, we performed additional equilibrium unfolding experiments by incubating 0.5 µM YME1L in the presence of urea concentrations ranging from 0 to 3 M. After overnight incubation at 25 °C, we incubated YME1L in the presence of 50 µM ANS (8-anilino-1-naphthalenesulfonic acid) for 5–10 min to allow for binding interactions to reach equilibrium. This experimental design allows for the observation of ANS:YME1L interactions after the unfolding equilibrium has been reached. Figure 4A demonstrates a 16% [urea]-dependent loss in ANS signal for conditions lacking urea versus 2–3 M urea in the absence of nucleotide as estimated by emissions at 480 nm. The observation of stable ANS emissions in the range of 1.5–3 M suggests the presence of a YME1L population that retains structure sufficient to support ANS binding in the absence of nucleotide, though the small decrease in F/F_o_ suggests a decreased concentration of structured YME1L species is present under these conditions. Incubation conditions that include the presence of 2500 µM ATP (Figure 4B) display similar signal-loss behavior to apo conditions. Given that altered tryptophan emissions are observed under these conditions (Figure 3), we must interpret this to suggest that a structural change does occur in the presence of nucleotide under these conditions, which causes a small decrease in the accessibility of ANS binding sites on YME1L.

### 3.3. Evaluation of YME1L—The Functional Impact of Urea on Nucleotide Binding and Unfoldase Activities

**Figure 5:**
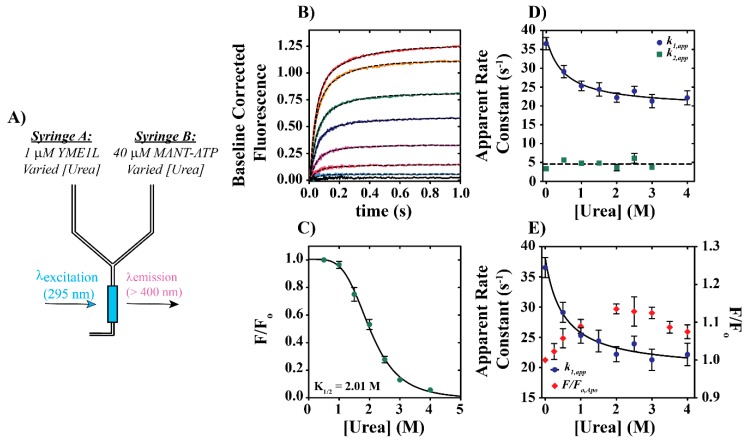
(**A**) Schematic representation of stopped-flow nucleotide binding experiments. Syringe A contains the indicated reagents, 1 µM YME1L and varied urea concentrations (indicated in text). Syringe B contains 40 µM MANT-ATP and varied urea concentrations. The urea concentration is identical in Syringes A and B for an individual experiment. The contents of the two syringes are rapidly mixed in the stopped-flow spectrophotometer and YME1L is excited at λex = 295 nm. Emissions are observed above 400 nm with a 400 nm-long pass filter. (**B**) Fluorescence time courses for YME1L binding of MANT-ATP. The black dashed lines represent NLLS fits to a double-exponential function,$\mathrm{Signal}=A_{1}\cdot \left(1-e^{-k_{1}\cdot t}\right)+A_{2}\cdot \left(1-e^{-k_{2}\cdot t}\right)$, where A_x_ and *k_x_* are apparent amplitude and rate constant terms. (**C**) Dependence of normalized fluorescence amplitudes observed at wavelengths above 400 nm in time courses presented in Figure 5B. The continuous line is the result of a NLLS fit to a cooperative model with *K_1/2_*, amplitude, and Hill coefficient equal to 2.01 ± 0.07 M, 1.02 ± 0.06, and 4.2 ± 0.5, respectively. (**D**) MANT-ATP concentration dependence of the apparent rate constants *k_1,app_* and *k_2,app_* observed at varied [urea] = 0, 0.5, 1, 1.5, 2, 2.5, 3, and 4 M. The continuous solid line is the result of a NLLS fit to a rectangular hyperbolic function with *K_1/2_* = 0.51 ± 0.06 M. (**E**) Overlay of F/F_o_ (Figure 3A) and *k_2,app_* (Figure 5D) versus [urea]. F/F_o_ and *k_2,app_* are represented as red diamonds and blue circles, respectively. All data represent average values determined from at least three independent experiments. Error bars indicate ± standard deviation.

The data presented above support a model wherein acute urea stress drives YME1L unfolding, followed by transition to a unique structure with an altered tryptophan solvent environment. This model does not require complete participation by the entire starting YME1L population, but instead allows for alterations in a smaller subset of the overall ensemble. However, the functional relevance of this the urea-treated ensemble remains unclear. From this, we asked: is YME1L equally capable of binding nucleotide across the range of urea concentrations examined here? To examine this question, we utilized a Fluorescence Resonance Energy Transfer (FRET)-based method wherein the nucleotide binding activity of YME1L was examined after urea treatment. We have previously applied this experimental design to study the nucleotide binding activity of YME1L under oxidative stress conditions [30]. Briefly, 1 µM YME1L was incubated at 25 °C in the presence of 0, 0.5, 1, 1.5, 2, 2.5, 3, 4, or 6 M urea for approximately 4 h prior to experimental use. Independent kinetic experiments have been performed reporting on time-dependent changes in tryptophan emissions and establish a minimum time of approximately 2 h to drive the refolded YME1L to equilibrium (Figure S1). Using urea-treated samples, we performed a series of stopped-flow kinetic experiments where 1 µM urea-treated YME1L was rapidly mixed with 40 µM MANT-ATP in the presence of urea and emissions were recorded as a function of increasing time (Figure 5A). Representative time courses shown in Figure 5B qualitatively indicate that nucleotide binding is dependent on [urea]. 

Representative time courses shown in Figure 5B reveal a urea concentration dependence in amplitude. Figure 5C shows a plot of F/F_o_ versus urea concentration calculated based on observed emissions relative to conditions lacking urea. The observation of a decreasing time course amplitude indicates that increasing [urea] promotes denaturation associated with loss of structure sufficient to promote nucleotide binding for most of the native YME1L ensemble. Therefore, we interpret the sigmoidal dependence of time course amplitude on [urea] presented in Figure 5C to represent an overall denaturation curve. Given our observation that YME1L unfolding is largely irreversible, we do not interpret denaturation as discussed here to be a reversible process. Instead, the midpoint of the plot shown in Figure 5C is intended to highlight the concentration of urea sufficient to yield a 50% decrease in nucleotide binding activity. NLLS analysis of the data shown in Figure 5C yields an estimate of *K_1/2_* = 2.0 ± 0.1 M. Reference to the equilibrium titration data presented for apo YME1L in Figure 3E suggests that the maximum peak position in F/F_o_ in equilibrium unfolding experiments may also serve as a predictor for the denaturation midpoint. Denaturation is defined here as the midpoint between the native and terminal states, which appears to roughly correspond with the urea concentration, promoting a maximum F/F_o_ as reported in Figure 3. Therefore, one would predict that saturating nucleotide concentrations above 500 µM ATP would yield increased denaturation midpoints equal to ~3 M urea. This is consistent with a role for nucleotide in stabilization against urea-dependent denaturation. Future work will be needed to examine this hypothesis using MANT-ATP binding strategies, since it will require additional analyses to differentiate contributions from [urea]-dependent unfolding and [ATP]-dependent competition with MANT-ATP to the overall decrease in apparent time course amplitude.

As reported previously, time courses reporting on YME1L binding of MANT-ATP are biphasic, consistent with at least two rate-limiting kinetic events [30]. All time courses shown in Figure 5B were subjected to NLLS analysis using a double-exponential function, thus providing estimates of relevant apparent first-order rate constants. Qualitative inspection of a plot of apparent rate constants, *k_1,app_* and *k_2,app_*, allows for multiple conclusions. The rate constant corresponding to the slow phase, *k_2,app_* (Figure 5D, green squares), is observed to fluctuate about a constant value equal to 4.6 ± 1.3 s^−1^, which is represented by the dashed line in Figure 5D. In contrast, the fast-phase rate constant, *k_1,app_*, exhibits [urea] dependence such that it decreases hyperbolically up to 4 M urea (Figure 5D, blue spheres). No MANT-ATP is observed to bind to YME1L in the presence of urea concentrations greater than 4 M. NLLS analysis of the relationship between *k_1,app_* versus [urea] estimates *K_1/2_* = 0.51 ± 0.06 M and an ~50% decrease in the apparent rate constant for nucleotide binding from 37 ± 2 s^−1^ to 22 ± 1 s^−1^ for [urea] = 0 M versus 4 M urea, respectively (Figure 5D). A decreased binding rate constant may be the consequence of nucleotide interactions with YME1L unfolding intermediates, wherein YME1L adopts a three-dimensional arrangement that is not optimized to support nucleotide binding comparable to wild-type YME1L. Alongside the decreased time course amplitudes, we expect that non-native YME1L species that populate after urea treatment represent only a subset of the overall YME1L population. 

Figure 5E illustrates an overlay of equilibrium protein unfolding data, F/F_o_, for apo YME1L (Figure 3A) and *k_1,app_* (Figure 5D) versus [urea]. This comparison is appropriate since YME1L included in Syringe A (Figure 5A) has been pretreated with urea in the absence of nucleotide. Thus, mixing of Syringes A and B is dependent upon the stability of apo YME1L. We note similarities between the *K_1/2_* = 0.51 ± 0.06 M reported in this section and the midpoint of the N to I transition equal to 0.6 M (Figure 3A). The overlay shown in Figure 5E suggests that the native and intermediate populations each bind nucleotide but do so with unique rate constants. Nucleotide binding observed over the range of 0–4 M urea may be due to interactions between MANT-ATP and a mixture of native/non-native YME1L species described by independent apparent rate constants. Moreover, the observation of no MANT-ATP binding by YME1L at [urea] > 4 M supports labeling the second transition observed in Figure 3 as a denaturation step.

**Figure 6:**
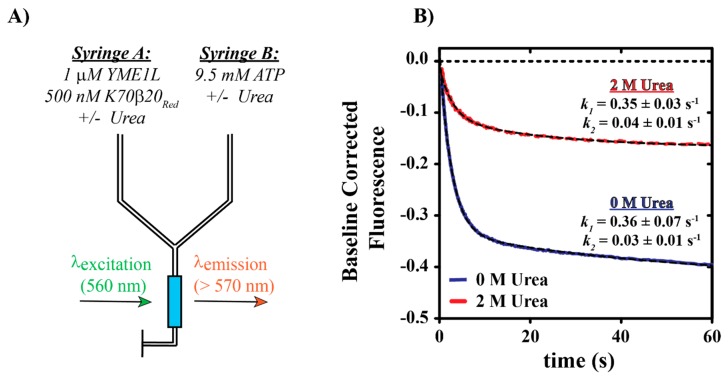
(**A**) Schematic representation of stopped-flow YME1L-catalyzed protein unfolding time courses. Syringe A contains the indicated reagents, YME1L and photoactivated K70β20 in the absence or presence of 2 M urea. Syringe B contains ATP to initiate YME1L-catalyzed protein unfolding as well as urea, if present in Syringe A. The unfolding reaction is initiated by rapid mixing of the contents of Syringes A and B in the stopped-flow spectrophotometer. K70β20 is excited at λ_EX_ = 560 nm and emissions are observed above 570 nm with a 570 nm-long pass filter. (**B**) Representative fluorescence time courses for YME1L-catalyzed K70β20 unfolding. Unfolding time courses are shown as solid blue or red dashed lines for experiments performed in the presence of 0 or 2 M urea, respectively. The black dashed lines represent NLLS fits to a double-exponential function,$\;\mathrm{Signal}=A_{1}\cdot \left(1-e^{-k_{1}\cdot t}\right)+A_{2}\cdot \left(1-e^{-k_{2}\cdot t}\right)$, where *A_x_* and *k_x_* are apparent amplitude and rate constant terms. All resulting estimates of apparent rate constants are indicated. All data have been repeated in at least three independent experiments. Error bars indicate ± standard deviation.

To determine whether [urea]-dependent decreases in nucleotide binding activity observed in Figure 5 translate into decreased YME1L-catalyzed protein unfolding activity, we performed additional stopped-flow fluorescence experiments, as schematized in Figure 6A. As presented, Syringe A of the stopped-flow spectrophotometer contains a solution of 1 µM YME1L previously incubated with or without 2 M urea as well as 500 nM photoactivated Kaede expressed with a C-terminal 70 amino acid tail containing the β20 degradation sequence (K70β20Red) [29]. Equilibrium unfolding experiments have been performed that confirm that no significant unfolding occurs for K70β20Red when incubated in the presence of 2 M urea (Figure S3). Upon mixing with 9.5 mM ATP in Syringe B, YME1L is expected to catalyze K70β20Red unfolding and subsequent degradation. Representative time courses are presented in Figure 6B. As expected, the mixing of the contents the Syringes A and B result in the observation of time-dependent loss of K70β20Red emissions at wavelengths above 570 nm. As previously reported, all time courses shown in Figure 6B are biphasic, where we have predicted the initial fast phase or secondary slow phase to represent a kinetic step related to protein unfolding or a step related to secondary unfolding cycles, respectively [30]. We previously reported apparent rate constants for the fast and slow phases, *k_1_* and *k_2_*, as 0.32 ± 0.01 s^−1^ and 0.023 ± 0.006 s^−1^, respectively [30]. NLLS analyses of time courses collected as schematized in Figure 6A estimate *k_1_* and *k_2_* as 0.36 ± 0.07 s^−1^ and 0.03 ± 0.01 s^−1^, respectively, in the absence of urea. In contrast, time courses collected in the presence of 2 M urea yield estimates of *k_1_* and *k_2_* as 0.35 ± 0.03 s^−1^ and 0.04 ± 0.01 s^−1^, respectively. Performance of one- or two-tailed Student’s t-test reveal no significant differences for kinetic parameters estimated in the presence or absence of 2 M urea. We note that the observation of a decreased nucleotide binding rate constant in Figure 5D is likely not captured in our unfolding assays due to K70β20Red unfolding being the rate-limiting step in these experiments. However, time courses collected in the presence of 2 M urea consistently exhibit mean amplitudes that are decreased approximately 3-fold relative to conditions lacking urea. These data are consistent with our analysis of nucleotide binding time course amplitudes wherein approximately 50% of the YME1L population is predicted to denature in the presence of 2 M urea under incubation conditions lacking nucleotide (Figure 5C). Decreased unfolding time course amplitudes presented in Figure 6B are consistent with a concomitant decrease in the concentration of functional YME1L complexes.

## 4. Discussion

The efficient maintenance of mitochondrial proteostasis is critical to the prevention of disease. Biochemical defects in mitochondrial protein function impact a wide range of activities that translate broadly into heterogeneous clinical outcomes. Common examples include respiratory failure, cardiomyopathy, sensorineural hearing loss, epilepsy, exercise intolerance, ptosis, dementia, Parkinson’s disease, Alzheimer’s disease, aging, cancer, etc. [41]. Stress conditions associated with varied disease states may contribute to mitochondrial dysfunction independent of genetic events. Cancer cells are commonly associated with increased mROS production leading to constitutive activation of growth factor receptors [42,43,44]. In contrast, chronic kidney disease is associated with increased oxidative stress and the production of toxic levels of uremic toxins that include urea, cyanate, p-cresol sulfate, indoxyl sulfate, etc. [22,23,24]. Such stress conditions may promote damage of mitochondrial biomolecules such as membrane lipids and critical protein components of the electron transport chain supracomplexes. Taken together, these examples demonstrate mitochondria as a potential site for concurrent stressors that include both chemical and oxidative stress. For this reason, rigorous examination of stress-induced changes in AAA+ protease behavior is necessary to fully understand how stress impacts the maintenance of mitochondrial proteostasis. Knowledge of the stability limits for enzymes involved in the maintenance of mitochondrial proteostasis will pave the way for the development of therapeutic compounds. 

### 4.1. Model for YME1L Denaturation

YME1L structure sufficient to support nucleotide binding and, presumably, downstream proteolytic activity appears to be stabilized in the presence of nucleoside triphosphate. Our rationale is based on two pieces of data presented here. First, equilibrium unfolding experiments indicate the presence of three distinct tryptophan solvent environments that populate depending on urea concentration. These species are resolved based on changes in YME1L tryptophan fluorescence at a given [urea] relative to conditions lacking urea. The observation of distinct YME1L populations based on changes in fluorescence must be the consequence of altered solvent environment for tryptophan residues, and, therefore, YME1L conformational differences promoted under the varied conditions examined here. We note that each tryptophan solvent environment identified here represents the population average, where each spectroscopically unique grouping is likely composed of multiple species. The position of maximum emissions corresponding to intermediate group formation is observed to shift with increasing nucleotide concentration such that 0, 20, 500, or 2500 µM ATP promote maximum intermediate species formation at 2.34 ± 0.05, 2.59 ± 0.006, 3.21 ± 0.06, or 3.27 ± 0.04 M urea, respectively. Secondary data derived from complementary ANS binding experiments performed in the presence of saturating nucleotide concentrations reveal only minor changes in YME1L:dye interactions as [urea] is increased to 3 M. Together, these data strongly indicate that the native YME1L tryptophan solvent environment is stabilized in the presence of 1-2 M urea and [ATP] ≥ 500 µM.

Equilibrium unfolding experiments do not conclusively demonstrate functional relevance. Instead, [urea]-dependent changes in tryptophan emissions may reflect the formation of non-functional unfolding intermediates, soluble aggregates, or alternate oligomeric states instead of enzymatically active conformations. For example, Figure 3 highlights apparent values for F/F_o_ similar to non-denaturing conditions when YME1L is incubated in the presence of [urea] > 5 M. Combined with stopped-flow fluorescence data that indicate a loss of nucleotide binding activity at [urea] > ~ 4 M (Figure 5), we interpret these combined data to suggest the formation of non-functional, yet soluble YME1L structures at high urea concentrations. Based on time course amplitudes (Figure 5B,C), we estimate the midpoint for this transition as 2.05 M, respectively. Thus, conditions including ~2 M urea that promote maximum F/F_o_ for apo YME1L (Figure 3A) also correspond to a midpoint between native and denatured YME1L populations. This observed correlation between a [urea]-dependent F/F_o_ maximum and denaturation midpoint suggests that the F/F_o_ may also be used to predict denaturation midpoint for conditions including nucleotide. However, further work will be required to examine this hypothesis. 

Our data clearly demonstrate that acute exposure to intermediate [urea] drives YME1L unfolding. Apparent time course amplitudes for nucleotide binding and protein unfolding activities indicate that unfolding occurs such that a significant population of non-functional YME1L exists as urea concentration is increased. Moreover, support for unfolding is derived from two observations. First, Figure 1D clearly demonstrates that pre-bound nucleotide undergoes dissociation upon mixing with urea. Experiments following tryptophan fluorescence on longer timescales indicate a similar trend. Increased urea concentrations promote a significant decrease in the concentration of YME1L hexamers that are functionally competent. Second, we observe decreased time course amplitudes in MANT-ATP binding experiments performed with YME1L samples treated previously with urea. No nucleotide binding is observed at urea concentrations greater than 4 M, which is consistent with [urea]-dependent stabilization of the denatured state. Decreased apparent rate constants for nucleotide binding at [urea] < 4 M are consistent with a structural rearrangement that may include promotion of a mixture of native, folding intermediate, and aggregated YME1L species. Additional studies will be necessary to fully characterize the kinetic mechanism of YME1L unfolding. 

We note ambiguity with regards to the events taking place at intermediate urea concentrations where increased tryptophan emissions are observed relative to conditions lacking denaturant. Treatment of YME1L with 2–3 M urea drives increased tryptophan emissions as well as a decreased rate constant describing MANT-ATP binding. Given our observation of poor YME1L unfolding reversibility in the presence of 2 and 6 M urea, we expect these conditions to populate a mixture of native and non-native YME1L species. Non-native species may represent a mixture of unfolding intermediates that retain nucleotide binding activity. However, we acknowledge that non-specific interactions may also be possible under such conditions. Regardless, MANT-ATP binding time course amplitudes suggest a sigmoidal decrease in the population of YME1L competent for nucleotide binding with increasing denaturant concentration with a midpoint at ~ 2M urea. Thus, we expect that the unknown contributors to binding in the presence of intermediate urea concentrations are not significantly populated at elevated urea concentrations. 

### 4.2. Comparison with Other ATP-Dependent Proteases

The FtsH-like family of proteases, including YME1L, is structurally distinct from other HSP100 proteases such as Lon or ClpAP through the presence of a transmembrane domain [45,46]. FtsH has been proposed to preferentially target previously destabilized protein substrates, which is consistent with its classification as a weak unfoldase [47]. Solubilized YME1L similarly exhibits weak unfolding power slightly greater than FtsH [28]. This is intriguing since available data indicate that *Escherichia coli* FtsH retains functionality in the presence of 3–5 M urea when supplemented with 2500 µM ATP [48]. We note that 2 M urea does represent a condition favoring 50% denaturation for apo YME1L, but saturating nucleotide concentrations likely shift this to [urea] > 3 M (Figure 3C,D). Nonetheless, YME1L is observed here to bind nucleotide and catalyze protein substrate unfolding in the presence of 2 M urea. Thus, FtsH family proteases may be capable of preferentially targeting stress destabilized proteins via a combination of transmembrane domain and nucleotide-dependent stabilization. In contrast, *Mycobacterium smegmatis* Lon protease has been reported to lose all ATPase and peptidase activity in the presence of 1 M urea as a consequence of urea-dependent oligomer dissociation [49]. Therefore, a potential stabilization role for the FtsH/YME1L transmembrane domain may reinforce structural integrity under conditions that promote loss of function for other ATP-dependent proteases involved in intracellular protein quality control and stress response. It is important to note that the data presented here do not suggest that the entire native YME1L ensemble will remain functional under stress conditions. Rather, these data suggest that a subset of the overall YME1L population may persist in a functionally active state. These YME1L complexes may be especially relevant under physiological stress conditions towards the maintenance of mitochondrial protein quality control.

## 5. Conclusions

The data reported here represent the first report of the stability limits for an ATP-dependent protease critical to the maintenance of protein quality control at the mitochondrial inner membrane. We have utilized multiple fluorescence-based techniques to clearly demonstrate that acute urea exposure drives YME1L denaturation. However, nucleotide binding appears to stabilize the overall YME1L population capable of nucleotide binding, leading to YME1L-catalyzed protein unfolding. These data are significant since they expand our knowledge of ATP-dependent protease resilience towards environmental stress. Future work will be needed to quantify the molecular mechanism underlying the observations reported here.

## Supplementary Materials

The following are available online at https://www.mdpi.com/2218-273X/10/4/656/s1, Figure S1, Figure S2, and Figure S3.

## Abbreviations

NTP	Nucleoside Triphosphate
ATP	Adenosine Triphosphate
AAA+	ATPases Associated with Various Cellular Activities
YME1	Yeast Mitochondrial Escape Protein 1
NLLS	Non-Linear Least Squares
LLS	Linear Least Squares
LEM	Linear Extrapolation Method
