# Supplementary material for: Fluorescence Methods Applied to the Description of Urea-Dependent YME1L Protease Unfolding
Source: Biomolecules. 2020 Apr 23;10(4):656. doi: 10.3390/biom10040656 (PMC7226517; doi:10.3390/biom10040656)
Supplement: Supplementary file 1 [file biomolecules-10-00656-s001.pdf]

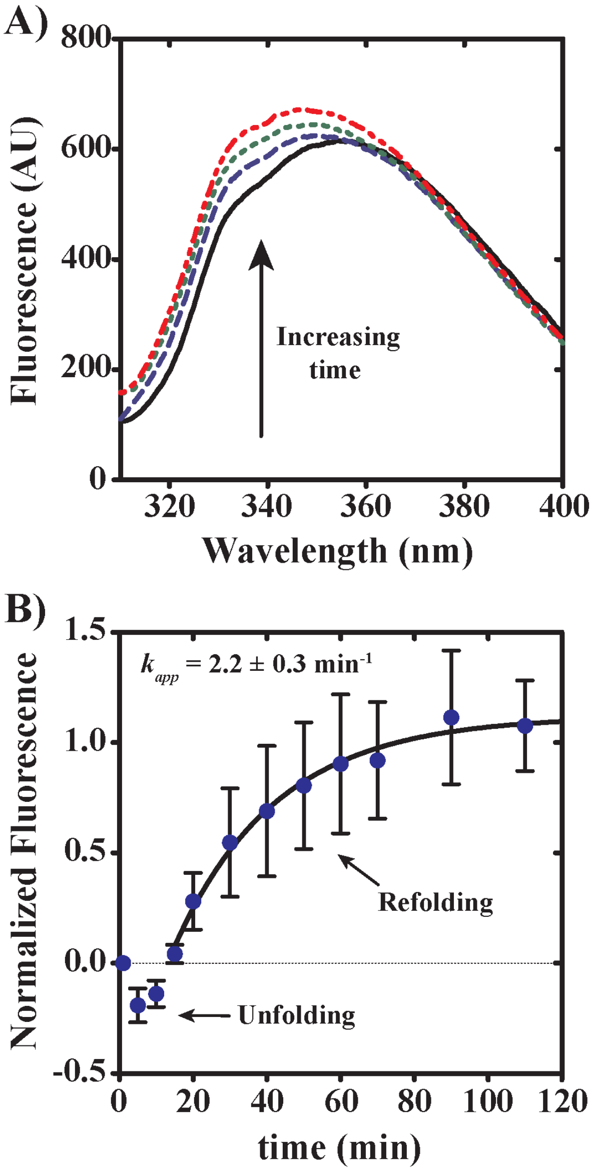

Figure S1. (A) Representative tryptophan emissions spectra collected in the presence of 2 M urea as a function of time. YME1L emissions spectra were collected using an excitation wavelength equal to 295 nm and emissions wavelength scanning from 310 to 400 nm. Samples were prepared by mixing 0.5 μM YME1L and 2 M urea in buffer H150 to initiate the reaction. Tryptophan emissions spectra were collected at defined time intervals across a total time span equal to 2 hours. Representative emissions spectra are shown that correspond to varied reaction time points equal to 5 (solid black line), 30 (blue dashed line), 90 (green dotted line), and 120 (red broken line) minutes. **(B)** Time-dependence of normalized fluorescence observed at 350 nm. The continuous line is the result of a NLLS fit of refolding phase data points to a single-exponential function with apparent rate constant equal to 2.2 ± 0.3 min^{-1}. All data represent average values determined from at least three independent experiments. Error bars indicate ± standard deviation.

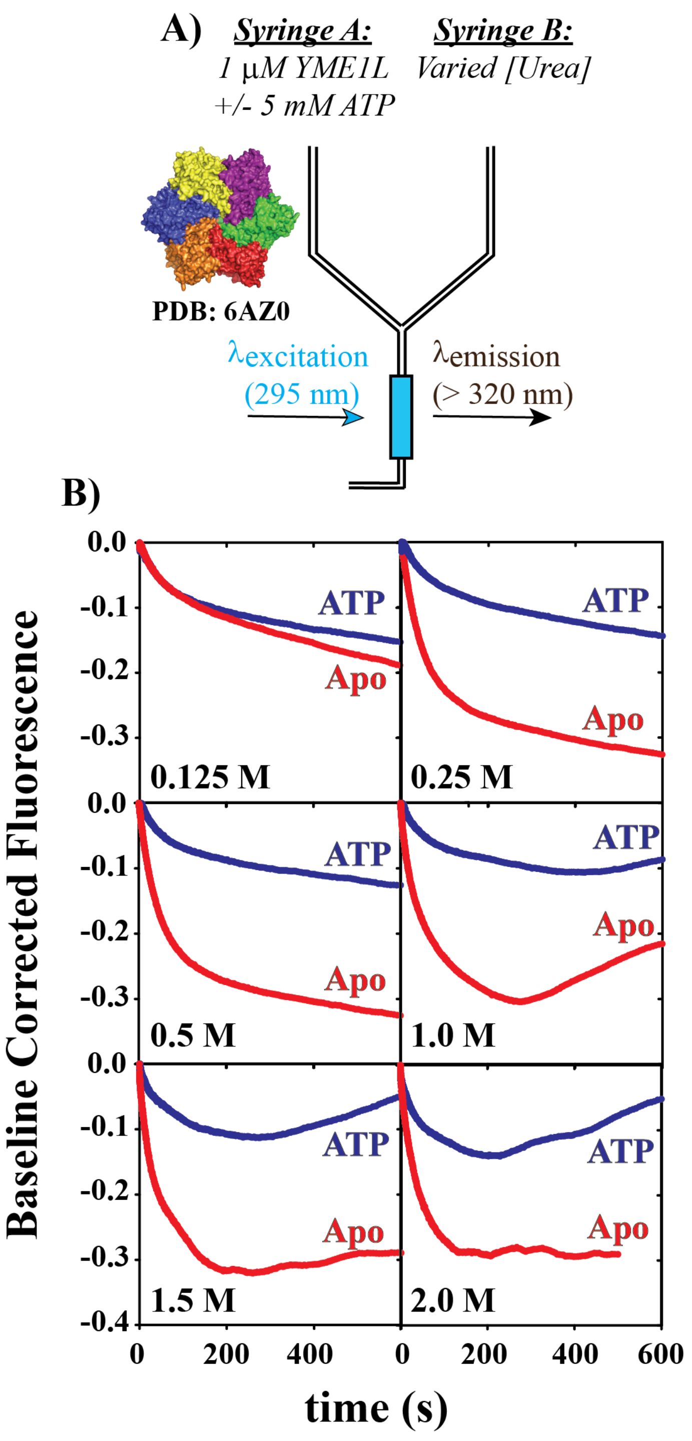

Figure S2. (A) Schematic representation of stopped-flow unfolding experiments reporting on tryptophan emissions. Syringes A and B contain 1 μM YME1L and indicated urea concentrations, respectively. The contents of the two syringes are rapidly-mixed in the stopped-flow spectrophotometer in the presence or absence of ATP. YME1L tryptophan residues are excited at λex = 295 nm. Emissions are observed above 320 nm with a 320-nm-long pass filter. **(B)** Representative fluorescence time courses reporting on tryptophan emissions for urea-dependent YME1L unfolding. All urea concentrations represent final mixing conditions. Experiments performed in the absence or presence of 2.5 mM (final mixing concentration) ATP are colored as red or blue lines, respectively. All data represent average values determined from at least three independent experiments.

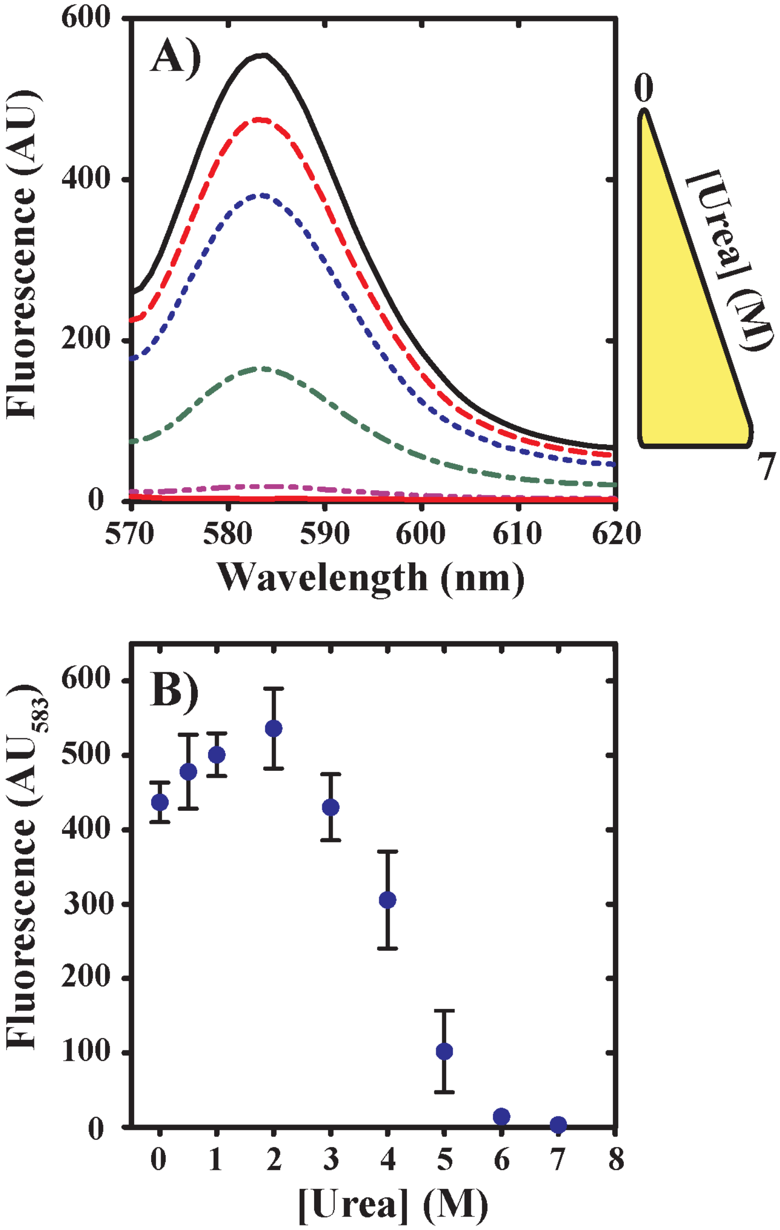

Figure S3. (A) Representative K70β20 emissions spectra collected in the presence of varied urea concentrations. Samples were prepared by overnight incubation at 25 °C of 0.5 μM K70β20 with varied urea concentrations. Samples were excited at 560 nm and emissions spectra collected by scanning from 570 to 620 nm. **(B)** Dependence of K70β20 fluorescence observed at 583 nm on [urea] All data represent average values determined from at least three independent experiments. Error bars indicate ± standard deviation.
